# Predicting triage of pediatric patients in the emergency department using machine learning approach

**DOI:** 10.1186/s12245-025-00861-z

**Published:** 2025-03-10

**Authors:** Manal Ahmed Halwani, Ghada Merdad, Miada Almasre, Ghadeer Doman, Shafiqa AlSharif, Safinaz M. Alshiakh, Duaa Yousof Mahboob, Marwah A. Halwani, Nojoud Adnan Faqerah, Mahmoud Talal Mosuily

**Affiliations:** 1 Pediatric Emergency Unit, Department of Emergency, College of Medicine, King Abdulaziz University, Jeddah, Kingdom of Saudi Arabia; 2 Faculty of Computing and Information Technology, King Abdulaziz University, Jeddah, Kingdom of Saudi Arabia; 3 Emergency Department, College of Medicine, King Abdulaziz University, Jeddah, Kingdom of Saudi Arabia; 4 Management Information Systems Department, College of Business, King Abdulaziz University, Jeddah, Kingdom of Saudi Arabia; 5 Department of Medical Microbiology, Faculty of Medicine in Rabigh, King Abdulaziz University, Jeddah, Kingdom of Saudi Arabia

**Keywords:** Canadian triage and acuity scale, K-Nearest neighbours, Support vector machine, Gaussian Naive Bayes, Decision tree, Random forest, Light GBM

## Abstract

**Background:**

The efficient performance of an Emergency Department (ED) relies heavily on an effective triage system that prioritizes patients based on the severity of their medical conditions. Traditional triage systems, including those using the Canadian Triage and Acuity Scale (CTAS), may involve subjective assessments by healthcare providers, leading to potential inconsistencies and delays in patient care.

**Objective:**

This study aimed to evaluate six Machine Learning (ML) models K-Nearest Neighbors (KNN), Support Vector Machine (SCM), Decision Tree (DT), Random Forest (RF), Gaussian Naïve Bayes (GNB), and Light GBM (Light Gradient Boosting Machine) for triage prediction in the King Abdulaziz University Hospital using the CTAS framework.

**Methodology:**

We followed three essential phases: data collection (7125 records of ED patients), data exploration and processing, and the development of machine learning predictive models for ED triage at King Abdulaziz University Hospital.

**Results and conclusion:**

The overall predictive performance of CTAS was the highest using GNB = 0.984 accuracy. The CTAS-level model performance indicated that SVM, RF, and LGBM achieved the highest performance regarding the consistency of precision and recall values across all CTAS levels.

**Supplementary Information:**

The online version contains supplementary material available at 10.1186/s12245-025-00861-z.

## Plain Language summary

A study used a dataset of records of ED patients to improve triage prediction accuracy using six machine learning models. The Gaussian-naive Bayes model was the most accurate, predicting triage levels at 98.4% of the time. However, SVM, Random Forest, and Light GBM outperformed each other in precision and recall, demonstrating that these models can enhance the consistency and accuracy of triage judgments in the ED.

**Supplementary Information:**

The online version contains supplementary material available at 10.1186/s12245-025-00861-z.

## Introduction

The efficient performance of an ED relies heavily on an effective triage system, which plays a crucial role in prioritizing patients based on the severity of their medical conditions and the urgency of treatment required [1]. The appropriate medical attention is critical, especially in life-threatening or time-sensitive emergencies where prompt intervention can significantly impact patient outcomes. One process crucial for the provision of these timely services in the ED is triage [2]. This is a process in which an initial clinical evaluation is conducted to select incoming patients who demonstrate an immediate demand for urgent care. The process typically uses a uniform scale to assess the severity of a condition before the physician assessment [3]. Many acuity-scoring systems have been developed to assess triage and the appropriate strategies for implementation in the ED environment [4]. One of the most commonly used models for triage is the CTAS, a widely adopted and standardized method used to categorize patients in EDs based on their clinical urgency [5]. The commonly used triage models is the CTAS, which is widely adopted internationally. However, in the United States, the Emergency Severity Index (ESI) is more frequently utilized. Both systems aim to categorize patients based on clinical urgency to optimize emergency department resource allocation [6].

However, traditional triage systems, including those using CTAS, may involve subjective assessments by healthcare providers, leading to potential inconsistencies and delays in patient care [7]. Different healthcare professionals may interpret patient’s symptoms differently, resulting in variations in triage decisions for patients with similar medical conditions. Such subjectivity can affect the accuracy of patient prioritization and resource allocation, potentially causing delays in critical cases or the unnecessary prioritization of less severe cases [8].

Traditional triage methods, such as the Emergency Severity Index and the Manchester Triage System, are prone to undertriaged and overtriaged, which can have a severe influence on patient outcomes and ED efficiency [9]. Undertriage, in which critically ill patients are incorrectly allocated lower acuity levels, can result in treatment delays and increased mortality risk. Overtriaged, on the other hand, causes lower-acuity patients to use key resources, which contributes to ED congestion [10]. Machine learning models are being developed to improve triage accuracy and speed patient flow.

In addition to the previous issues in traditional triage methods, we observed a notable lack of consulting retrospective data records of ED patients to reconsider decision-making [11]. One primary reason for this is the sheer size and complexity of available data. EDs often handle a large number of patients with diverse medical conditions, resulting in the accumulation of vast amounts of historical patient data over time [12]. We observed the lack of standardized methods and tools for analyzing this retrospective data which further complicated the decision making. The absence of robust data analytics platforms and expertise may also contribute to the underutilization of retrospective data in CTAS-based decision-making [13, 14].

Advancements in machine learning have led to the development of predictive models that often outperform traditional statistical methods in diagnosis and prognosis. Several ML models have demonstrated superior accuracy in predicting critical care outcomes, such as ED to intensive care unit (ICU) transfers and in-hospital mortality, compared to conventional screening tools like the Modified Early Warning Score, National Early Warning Score, and Sequential Organ Failure Assessment [15, 16]. In radiology, ML-based radiomics models have exceeded human performance in detecting subtle abnormalities that are often imperceptible to the naked eye. The practical implementation of the proposed model depends on its computational efficiency, seamless integration into clinical workflows, and clinician acceptance. However, models like Random Forest and SVM present interpretability challenges, which may hinder trust and adoption in emergency settings [17]. To enhance transparency, we utilized SHAP (Shapley Additive Explanations) to identify key clinical variables influencing triage predictions and LIME (Local Interpretable Model-Agnostic Explanations) to provide case-specific interpretations for decision support. Clinician acceptance can be strengthened through user testing of SHAP/LIME outputs, integration with electronic health record (EHR) systems for streamlined decision-making, and validation against expert physician assessments to ensure reliability [18, 19].

To address these challenges and improve the effectiveness of the triage process using retrospective data, this study proposes a Machine Learning (ML) approach for triage prediction at King Abdulaziz University Hospital (KAUH). The wealth of patient data available to hospitals via the ED’s systems is unmatched and can be used to create many applications that are useful in the ED context and can improve the management of the ED department and the allocation of hospital resources in a useful way.

### Literature review

Literature reported that the triage health care provider assessment in emergency care systems is difficult due to the growing number of patients and congestion. Traditional triage methods have issues with patient sorting and human error, which can risk patients’ lives. Machine learning (ML) technology can automate the triage decision-making process, resulting in more accurate and faster patient evaluations [20]. ML has demonstrated superior performance in predicting hospitalization and critical-care outcomes compared to reference triage models, possibly addressing overcrowding, enhancing health services, and lowering morbidity and death rates [21]. The literature reported the accuracy of a three-level triage system performed by triage nurses, and emergency medicine doctors in an ED. Data from 500 patients, including vital signs, primary complaints, age, and gender, were analyzed. Only 23.8% of patients received identical triage categorizations across all evaluators. Compared to emergency medicine doctors, triage nurses demonstrated slight overtriaged (6.4%) and undertriaged rates of 3.1% for yellow-coded and 3.4% for red-coded patients. Among AI models, demonstrated the highest accuracy but still undertriaged 26.5% of yellow-coded and 42.6% of red-coded patients. Given the significant undertriaged rates, AI models are not yet suitable for independent triage in emergency settings, requiring further optimization before clinical implementation [22].

The study conducted by Dugas et al. describes a computer-based electronic triage system (ETS) that optimises patient acuity distribution based on critical patient outcomes. The study evaluated the ETS to the Emergency Severity Index (ESI) in terms of patient distinction, outcomes, inpatient hospitalization, and resource utilization. The ETS dispersed patients more equally, identified patients with composite outcomes, and enhanced resource utilization. The ETS demonstrated a small improvement in patient distinction [23]. The study reported that e-triage more reliably detects ESI level 3 patients and emphasizes the potential of predictive analytics. The system predicts the requirement for critical care, emergent surgical intervention and inpatient hospitalization via a random forest model. At both EDs, e-triage outperformed the ESI in identifying clinical patient outcomes. E-triage detected more than 10% of ESI level 3 patients who needed up-triage and were at risk of critical care or an emergent surgical intervention [24].

To enhance patient triage in pediatric ED through the use of machine learning (ML) the study used a huge dataset of 189,718 patient visits over three years, with 9271 instances (4.98%) not hospitalized. Four machine learning models were tested: Deep Learning, Random Forest, Naive Bayes, and Support Vector Machines. The results demonstrated that ML prediction models trained on clinical outcomes performed better in triage than the present rule-based expert system. The study is among the first to use machine learning for pediatric ED triage [25]. A research in a Korean tertiary hospital attempted to predict early critical interventions (CrIs) for critically ill patients. The Extreme Gradient Boost (XGBoost) prediction model was utilized in the study, which had 137,883 patients. The model revealed that higher CrIs were related to worse ED outcomes. The CrIs model was incorporated into the site’s electronic medical record, allowing emergency physicians to propose early therapies [26]. Another study demonstrated that machine learning can reliably predict Korean triage Acuity Scale (KTAS) levels during triage reported to develop and compare machine learning models for predicting KTAS levels in ED. The random forest and XGBoost models exhibited the greatest AUROC, followed by clinical data-trained models [27].

Logistic regression is a prominent reference model for clinical triage prediction, however current research indicates XGBoost and deep neural networks as older techniques in terms of predictive accuracy. XGBoost is one of the best-performing triage classification models, whereas DNNs detect complicated non-linear patterns in clinical data. However, these models confront computational complexity and transparency issues, prompting more study into their incorporation into ED procedures [10].

The literature emphasizes the utility of machine learning in predicting clinical outcomes and dispositions in EDs. The reported research presented pediatric patients aged 18 years or younger who visited the ED Lasso regression, random forest, gradient-boosted decision tree, and deep neural network models were used. Their findings revealed that all machine learning algorithms had better discriminative ability for critical care and hospitalization, with fewer critically ill children undertriaged and fewer children overtriaged who did not require inpatient management. The decision curve study revealed that machine learning models provided a larger net benefit over a wide variety of clinical criteria [28]. The Manchester Triage System (MTS), a five-level triage system in Europe, categorizes patients based on symptom severity and urgency, aiming to prioritize timely care and optimize resource utilization, similar to CTAS and ESI. The effectiveness of ML-based prediction in executing the MTS was investigated utilizing data from Kepler University Hospital, in which RF and Neural Networks (ANN) were trained on the data to predict patient outcomes, such as discharge or admission for observation or intensive care. The results indicated that both RF and ANNs outperformed the other models in tasks, such as ward observation admission, intensive care admission, and 30-day mortality prediction [29].

ML-based triage prediction is dependent on the selection of relevant clinical parameters. Recursive feature removal and principal component analysis are two techniques that optimise feature sets during training. Cross-validation is a validation strategy that ensures model dependability. Resampling methods such as Synthetic Minority Over-sampling Technique (SMOTE) and ADASYN increase model performance at under-represented CTAS levels. Future study should investigate how these methods affect real-world triage accuracy and clinical decision support [10].

Previous research used machine learning to predict triage inside the CTAS framework. Hall et al. (2023) created an ML-based acuity score prediction model for virtual care environments [30], while Chen et al. (2023) used deep neural networks to predict important outcomes in ED patients [31]. However, these research were largely concerned with single prediction models rather than a comparative analysis of numerous ML algorithms. Furthermore, only a small amount of research has been conducted on using retrospective CTAS data analysis to improve triage accuracy in clinical settings. Our work fills this gap by creating and testing six ML models (KNN, SVM, DT, RF, GNB, and Light GBM) using a large retrospective dataset from King Abdulaziz University Hospital (KAUH), resulting in a complete health care provider assessment of ML performance in triage prediction.

Even systematic reviews reported that the implementation of ML models can contribute to better predictions of acuity scales. Literature focused on predicting patient’s need to access intensive care services. Among the ML models implemented in the reviewed studies are gradient boosting, logistic regression, neural networks, support vector machines, and random forests. The results indicated that Gradient Boosting, Logistic Regression, ANN and SVM demonstrated high performance in terms of accuracy ranges compared to other models [32].

Recent comprehensive evaluations have underlined the expanding importance of ML in emergency triage prediction. Sánchez-Salmerón et al. (2022) conducted a comprehensive study of ML approaches used in emergency triage, highlighting their potential to enhance decision-making and patient flow [3]. Similarly, Miles et al. (2020) examined ML-based risk prediction models and concluded that, while ML improves triage accuracy, model interpretability and incorporation into clinical practice remain problems [33]. More recently, Porto (2024) did a comprehensive study on the use of machine learning and natural language processing (NLP) in triage, revealing key research needs in data standardization and real-time deployment [10]. These studies underscore the need for more research to improve ML-based triage systems, particularly in pediatrics emergency situations.

Generally, these studies explore various ML models, and as the results indicate, there is no single model that outperforms every implementation of triage prediction, which means that the context of the study, type of data features, and size contribute to model performance. However, these studies helped in selecting the models that we wanted to explore in our experiment, including the SVM, RF, and KNN.

### Rationale of the study

By leveraging ML techniques and algorithms, this study aimed to evaluate six ML models that objectively assessed patient’s acuity levels based on their clinical data. By minimizing subjectivity, these models seek to provide more accurate and consistent triage decisions, resulting in improved patient flow and optimized resource allocation within the ED. Therefore, the study trained six robust ML models for triage prediction in hospital EDs using the CTAS framework based on a large retrospective dataset from King Abdulaziz University Hospital and evaluate the overall accuracy of ML models for triage prediction on a dataset to determine the model with the highest accuracy and to evaluate the performance of ML models in the prediction of each CTAS level using the metrics of F1-Score, Precision, and Recall.

## Methodology

### Study design and settings

A single-centred retrospective study was conducted at King Abdulaziz University Hospital (KAUH) after ethical approval was granted from the relevant party. A retrospective dataset comprises pediatric patients admitted to the ED between September 2021–2023. The personally identifiable data was not part of the dataset.

### Data collection

The data were randomly collected via the official KAUH hospital information system (Phoenix), arranged by the hospital administration and based on an ethical approval agreement. Data extraction was carried out by certified healthcare professionals and clinical researchers with experience working with electronic health records (EHR). The team consisted of senior physicians, data analysts, and research coordinators who had been educated in medical coding, data protection standards (GDPR/HIPAA), and statistical analysis. Their expertise guaranteed reliable data gathering, adherence to ethical principles, and conformity with institutional and regulatory requirements. The extracted dataset represented the hospital records of ED patients. The dataset included multiple features that were filtered to include only relevant features for our study. The dataset present the hospital records of pediatric patients (birth–14 years old) who visited the ED. Initially, multiple features were included, but only those relevant to the study were retained. Patients were excluded if their medical records were incomplete or contained missing critical variables necessary for accurate classification, such as triage level, chief complaints, or vital signs. Additionally, individuals who were deceased upon arrival or within the ED were excluded to maintain a focus on triage assessment rather than mortality prediction. Missing values were addressed using median imputation for numerical variables, ensuring data completeness without arbitrary removal. Data cleaning involved imputing or removing missing values, correcting implausible entries, eliminating duplicates, and standardizing categorical variables. Outliers were identified using box plots, while numerical features were normalized, and categorical data were encoded for consistency. After preprocessing, the final dataset consisted of 7,125 records of ED patients, with the included features detailed in the tabulation (Table 1).

**Table 1:** KAUH dataset features

Feature Name	Feature Description	Type
ID	The patient’s anonymized ID number	Identifier
PAT_SEX	Sex of the patient, either male or female	Categorical
AGE	A continuous variable from 0–14	Numerical
SBP	Systolic blood pressure	Numerical
DBP	Diastolic blood pressure	Numerical
HR	Heart rate	Numerical
RR	Respiratory rate	Numerical
BT	Body Temperature	Numerical
Saturation	Oxygen Saturation (SPO2)	Numerical
CTAS	Canadian Triage and Acuity Scale (five levels)	Categorical

### Data exploration and processing

To draw information about the quality and breadth of the data, we performed Exploratory Data Analysis (EDA), which resulted in basic descriptive statistical attributes. The descriptive statistics of the data represented a young demographic with a mean age of 5.6 years. Blood pressure readings demonstrated the average of SBP; 118.256 mmHg and DBP; 73.693 mmHg. The heart rate (HR) was 80.355 bpm, with a range from 50 to 109 bpm respectively, with normal distribution. The ranges for these features (80–159 mmHg for SBP and 40–109 mmHg for DBP) were consider normal. Similarly, the respiratory rate (RR) has an average of 28.305 breaths per minute, which was relatively higher but justifiable considering that it can be normal for young children. In contrast, the body temperature (BT) and SPO_2_ values were slightly below the recorded averages of 37 °C (98.6 F) and 95–100% respectively. The data revealed that the majority of patients were classified as CTAS Level 3, creating an unbalanced dataset in which this group accounted for the majority of instances. This mismatch may cause biases in model performance since the model may favour the dominant class (CTAS Level 3) over the under-represented classes (CTAS Levels 1, 2, 4, and 5) (Table 2). The distribution of the dataset was substantially biased towards CTAS Level 3, accounting for a considerable proportion of the cases.

**Table 2:** Descriptive statistics of the data

Feature	count	mean	SD	min	10%	25%	50%	75%	90%	95%	99%	max
ID	7125	3563	2056.955	1	713.4	1782	3563	5344	6412.6	6768.8	7053.76	7125
AGE	7125	5.591	4.103	1	1	2	4	9	12	13	14	14
SBP	7125	118.256	13.318	80	101	108	118	129	136	139	147	159
DBP	7125	73.693	10.75	40	60	66	74	82	87	89	98	109
HR	7125	80.355	8.654	50	70	74	80	86	91	96	102	109
RR	7125	28.305	3.143	20	24	26	28	31	33	33	35	37
BT	7125	35.792	0.35	34.505	35.39	35.593	35.773	35.957	36.268	36.43	36.817	36.997
Saturation	7125	92.213	1.946	85	90	91	92	94	94	96	97	99
CTAS	7125	2.91	0.636	1	2	3	3	3	4	4	4	5

The study used [SMOTE/ADASYN] to address class imbalance, specifically the overrepresentation of CTAS Level 3, by synthesizing minority class samples. SMOTE was applied with k = 5 nearest neighbors to generate new instances for CTAS Levels 1, 2, 4, and 5. Oversampling was performed on the training set to prevent data leakage, and stratified sampling ensured proportional representation of each CTAS level during cross-validation. This balanced the proportion of each CTAS level across subsets during training and testing, minimizing the risk of amplifying imbalance.

In the data-processing step, we split the dataset into training and test sets at a ratio of 70/20 (70% training, 10% validation, and 20% testing). This split step included a one-hot encoding step in which categorical features (PAT_SEX and CTAS) were transformed into numerical features.

### Developing ML predictive models of ED triage in KAUH

For the prediction task, we selected six ML models to classify the dataset samples based on CTAS levels [1–5]. Each of these models has its value or contribution to the classification task, and we wanted to experiment with all of them to align the value with the actual prediction outcome.

The Light Gradient Boosting Machine (LGBM) had been demonstrated to perform well in clinical prediction tests and was useful in triage applications. It employs a number of learning methods, including KNN, SVM, GNB, DT, RF, and gradient-boosting [3, 34, 35]. Tree-based models, such as Random Forest and Light GBM, regularly produce good predicted accuracy in medical categorization challenges. SVM was appropriate for high-dimensional data in triage scenarios. Naïve Bayes was a computationally efficient benchmark for real-time applications, while Decision Trees provide interpretability in clinical decision-making. These models achieved an appropriate mix between predictive performance, computational efficiency, and interpretability, making them viable options for real-world implementation [35–37].

#### Algorithms

The constructed models used the following algorithms.

- **KNN (K-Nearest Neighbors)** is a non-parametric algorithm that utilized for classification tasks, where it identifies the ‘k’ nearest data points to a predefined instance based on a distance metric (e.g., Euclidean distance). The majority class among these neighbours was determined as the prediction for the sample. KNN was suitable for ED triage classification in cases where the decision boundary is locally smooth and the dataset is not that large [38].
- **SVM (Support Vector Machine)** that’s a supervised learning algorithm used often for prediction and regression tasks. It locates the hyperplane that ideally separates the data points of different classes while maximizing the margin between them. SVM reported to be effective for our project because it captures the complex relationships between features [39].
- **GNB (Gaussian Naive Bayes)** considered a probabilistic classifier which assumes that features are independent. Each class’s conditional probability was computed based on the features, and the class with the highest probability was selected as the prediction. GNB is suitable for ED triage prediction because its features are conditionally independent compared with the CTAS level [40].
- **Decision Tree Classifier (DTC)** a tree-based classifier that partitions the feature space recursively based on feature values. It outputs its decisions using a tree-like structure, in which each internal node represented a decision based on a feature, and each leaf node represented a class label. DTC can be used for triage prediction because it is easy to interpret and visualize [41].
- **RF (Random Forest)** reported as an ensemble learning method that constructs multiple decision trees during training and combines their results to improve accuracy. This model can be useful for avoiding overfitting [42].
- **LGBM (Light GBM)** a gradient-boosting framework capable of building multiple decision trees sequentially to reiterate the errors of the previous trees and correct them. This algorithm were effective in contexts where high predictive accuracy and computational efficiency are required [43].

### Training the models

The next stage in our prediction pipeline involved developing a framework that aided in selecting the models and providing parameters for the optimization of these models. The objective were to execute a classification or prediction task based on the CTAS levels in the dataset. It implements a combined process that initially performs a grid search to tune the hyperparameters of the RF and LGBM models and then trains the other four KNN, SVM, DTC, GNB, and optimized RF and LGBM classifiers. Hyperparameters adjustment was carried out to improve model performance, notably for Light GBM and Random Forest. We used a grid search/random search/bayesian optimization strategy to systematically investigate the best combination of hyperparameters. Key parameters for Light GBM, such as the number of leaves, learning rate, and maximum depth, were tuned to strike a compromise between model complexity and generalization. To prevent overfitting while retaining robust performance, we optimised Random Forest’s number of estimators, maximum depth, and minimum samples per split. The tuning procedure was tested using k-fold cross-validation (e.g., 5-fold or 10-fold), to ensure that the chosen hyperparameters enhanced model stability across multiple data splits.

### Evaluation metrics

When the training step was executed, the evaluation step commences using an iterative loop through each model to predict the CTAS levels based on a test set and calculate the accuracy of these predictions compared to the true labels. The models were evaluated against the recorded CTAS scores assigned by triage nurses at the time of patient assessment, which served as the ground truth for comparison. Since CTAS assignment inherently involves clinical judgment, the ‘true labels’ in this study reflect expert-documented triage decisions rather than an independent gold standard. Thus, model predictions approximate the decision-making patterns of human triage personnel rather than an absolute measure of acuity. This highlights the potential for ML models to enhance consistency and decision support in triage.

Accuracy was computed as the ratio of correctly classified samples (true positives and true negatives) to the total number of samples in the original data. The results of this computation provide an overview of the performance of the model by calculating the percentage of correct predictions.

$$\:Accuracy=\frac{\text{T}\text{r}\text{u}\text{e}\:\text{P}\text{o}\text{s}\text{i}\text{t}\text{i}\text{v}\text{e}\text{s}+\text{T}\text{r}\text{u}\text{e}\:\text{N}\text{e}\text{g}\text{a}\text{t}\text{i}\text{v}\text{e}\text{s}}{\text{T}\text{o}\text{t}\text{a}\text{l}\:\text{P}\text{r}\text{e}\text{d}\text{i}\text{c}\text{t}\text{i}\text{o}\text{n}\text{s}}$$

In addition to the accuracy, metrics such as precision, recall, and F-score were computed, and confusion matrices were generated to discuss the results.

## Results

### Comparison of ML models and their predictive results

**Fig. 1:**
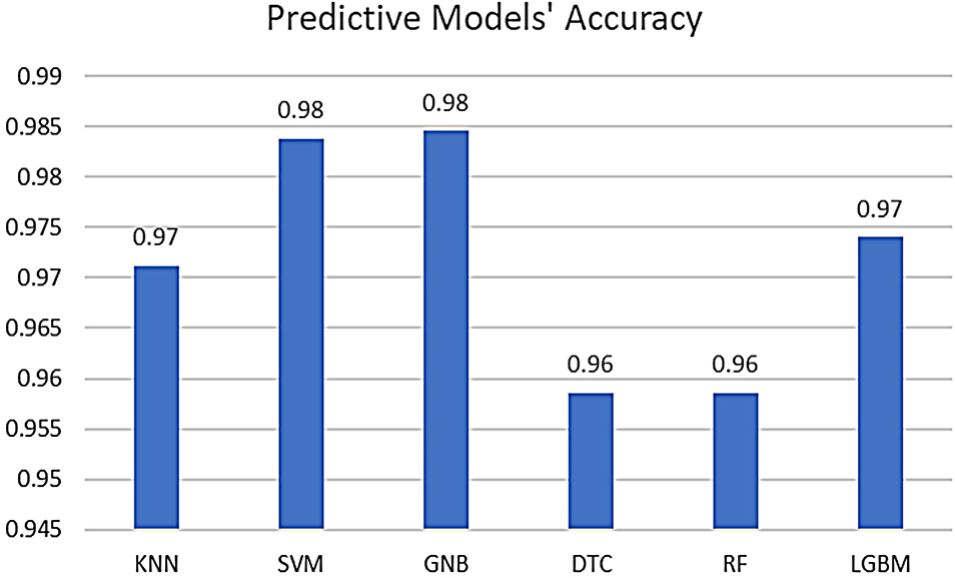
Comparison of ML models’ overall performance

Running the six models on the KAUH dataset as per the parameters specified previously resulted in the overall good performance of these models on the dataset. As indicated in Fig. 1, all six algorithms performed relatively well, with an accuracy of above 0.955. However, the best-performing model in the prediction of CTAS levels was dependent on the GNB algorithm, with an accuracy of 0.984, followed closely by the SVM model, with an accuracy value of 0.983. Overall, these results provide valuable insights into the strengths and weaknesses of each algorithm in accurately segmenting data, guiding further refinement and optimization of the segmentation process.

**Fig. 2:**
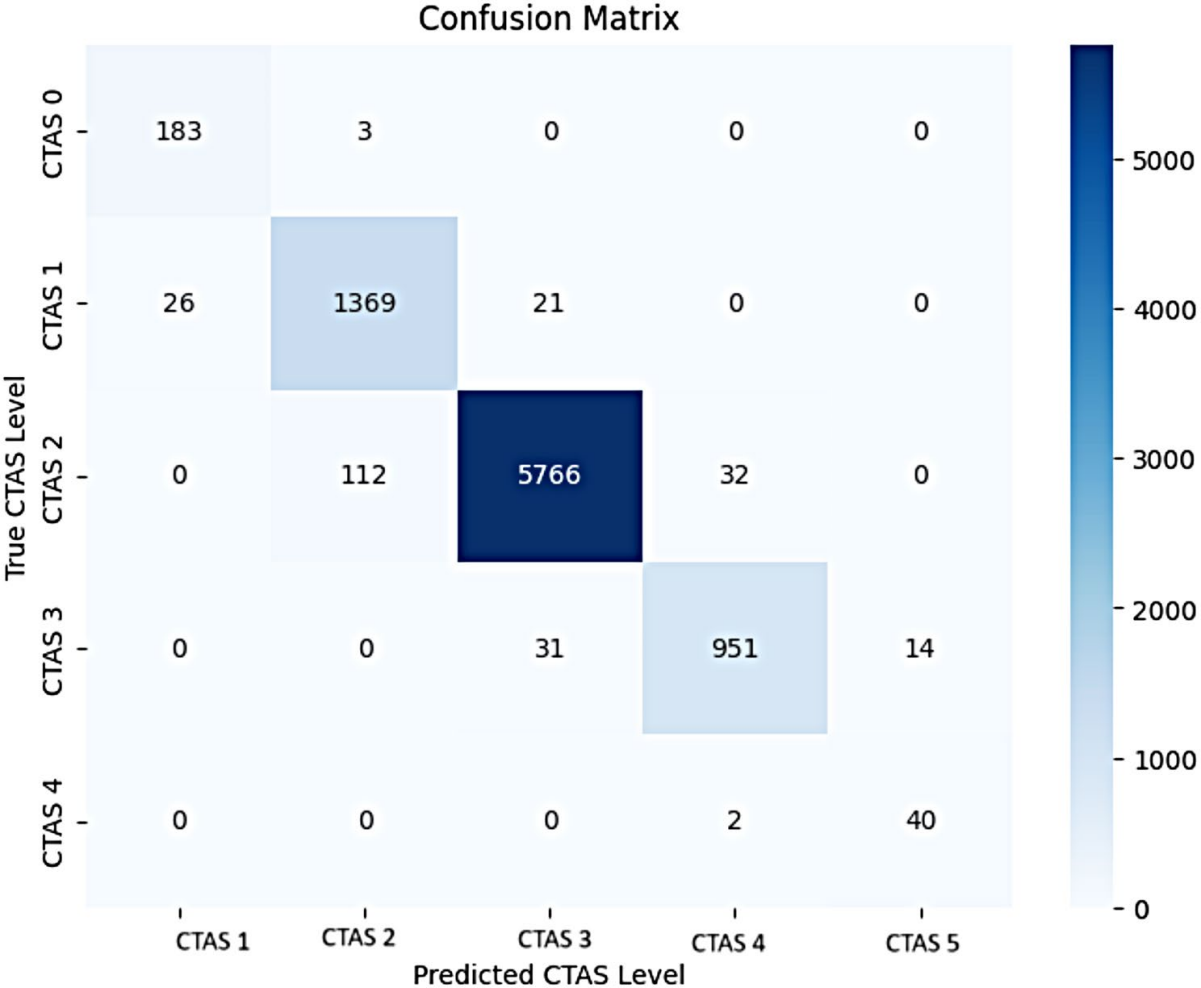
Confusion matrix of combined prediction results for all models

Examination of the confusion matrix sheds light on further considerations. The matrix indicates that the models are collectively capable of classifying CTAS 2, but they demonstrated inconsistencies when attempting to classify the extreme levels of CTAS (CTAS 1 and CTAS 5) because there was a lower occurrence of false positives and false negatives for these two levels. This could be attributed to the small sample sizes at these two levels. It was also notable that instances of misclassification occurred between closely adjacent CTAS levels (Fig. 2).

### Models performance classification at the CTAS level

**Fig. 3:**
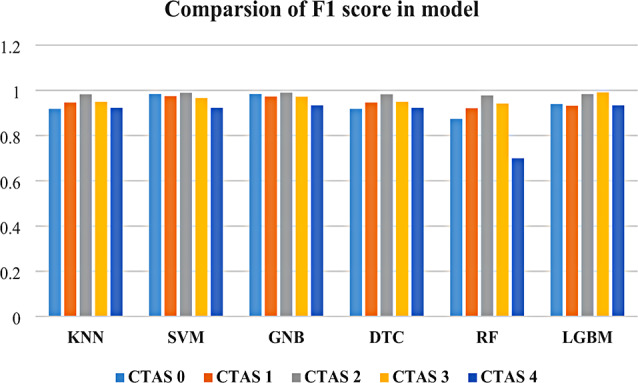
Comparison of F1 score across models for each CTAS Level

Next, we analyzed the models’ performance on the classification of each CTAS level by examining other evaluation metrics such as Precision, Recall, and F1-score. The evaluation results for each model, and the best-performing model in terms of individual-level prediction was the GNB model, with a mean F-score of 97 (Fig. 3) (Suppl. Tables S1-S6). Among the results, the aspects that captured our attention were those related to the highest F-score value achieved by each model per CTAS level. We noticed that five of the six models achieved the highest F-scores for predicting a CTAS score of 3. These models were the SVM, RF, KNN, GNB, and DTC. The LGBM model achieved a high F-score on CTAS 4.

Another aspect pertains to how the models were performed in terms of their precision and recall of the documented values. According to these results, both SVM models consistently outperformed the other models in terms of accuracy and robustness, achieving a lower misclassification across all levels. Both RF and LGBM achieved relatively high precision and recall scores for CTAS 3 and CTAS 4 predictions. However, GNB and DTC performed lower at certain CTAS levels considering their precision and recall values.

The overall diagnostic accuracy of the predictive models in classifying patients based on their actual CTAS score was high, with an accuracy of 97.25% (95% CI: 96.84–97.62%). The sensitivity, which measures the model’s ability to correctly identify actual positive cases (i.e., patients who should receive a higher triage score), was 97.69% (95% CI: 97.22–98.10%), indicating strong performance in detecting critical cases. Similarly, the specificity, reflecting the model’s ability to correctly identify actual negative cases, was 96.36% (95% CI: 95.53–97.08%), suggesting a low rate of false positives. The positive predictive value (PPV) of 98.18% (95% CI: 97.78–98.52%) indicates that most patients predicted as high-acuity were indeed high-acuity cases, while the negative predictive value (NPV) of 95.39% (95% CI: 94.51–96.14%) demonstrated the model’s reliability in ruling out non-urgent cases. These results suggest that the predictive models provide a robust and reliable classification of CTAS scores, supporting their potential use in enhancing triage accuracy in emergency settings (Table 3).

**Table 3:** Overall diagnostic accuracy of models with actual CTAS score

Overall model findings	CTAS at ED		Total
	Actual positive	Actual negative	
Predictive Positive	4651	86	4737
Predictive Negative	110	2278	2388
Total	4761	2364	7125
**Diagnostic accuracy**	**Value**	**95% CI**	
Sensitivity	97.69%	97.22–98.10%	
Specificity	96.36%	95.53–97.08%	
Positive Predictive Value	98.18%	97.78–98.52%	
Negative Predictive Value	95.39%	94.51–96.14%	
Accuracy	97.25%	96.84% to 97.62	

## Discussion

Increasing overcrowding in EDs, and extended lengths of stay necessitate more efficient triage evaluations. Machine learning (ML) algorithms offer a promising solution by automating tasks, analyzing complex data, and improving triage predictions. Leveraging electronic health records (EHRs), these models can identify patient symptoms, retrieve medical data, and forecast clinical needs while capturing intricate interactions [44].

Recent research has looked at novel ways for enhancing ML-based triage and ED management, such as a new feature engineering methodology for forecasting patient arrivals in EDs. This strategy can improve model performance, optimised resource allocation, and enable real-time decision-making. Future study should look at leveraging these developments to CTAS-based triage systems to improve their predictive powers [45].

This study provided a detailed evaluation of six ML models for predicting Canadian Triage and Acuity Scale (CTAS) levels in an emergency setting. Key findings highlight the variability in model performance, emphasizing the importance of algorithm selection in determining predictive accuracy. Factors such as model complexity, handling of nonlinear relationships, and generalizability to new data influence effectiveness. Beyond predictive performance, practical considerations—including computational efficiency, interpretability, and seamless integration with existing healthcare systems—are crucial for real-world implementation.

The study investigated by Georgios Feretzakis et al., reported the application of artificial intelligence in emergency care, focussing on demographics, coagulation tests, and biochemical markers used during hospitalizations and demonstrated AI’s potential to improve healthcare services in emergency medicine [46]. Another study suggested algorithms performed well in predicting hospital admissions for ED patients, with F-measure and ROC Area values. These models have advantages such as ease of use, availability, and yes/no outcomes, and low cost. The clinical consequences might shift away from traditional decision-making and towards more advanced models, and the study could influence the future of emergency care. Implementation in pragmatic ED trials is warranted [47].

ML approaches can increase predictive triage abilities in a variety of illnesses, helping clinicians to make better judgements and tailor therapy. ML-based triage models have proven to be more accurate predictors of critical-care outcomes and hospitalization ensure appropriate patient allocation and help to make better decisions [48]. These skills can help to enhance patient routes, manage hospital resources, save expenses, and minimise wait times and length of stay (LOS) so it can assist addressing overcrowding, enhance healthcare services, and lower morbidity and death rates.

Complex models such as SVM or ensemble models such as RF provided more accurate results for CTAS-level predictions, which were consistent with the model’s performance in previous research that affirms its potential as a reliable algorithm for triage prediction in the ED [49]. In the current study, the SVM and Random Forest models provided strong performance in terms of precision and recall, but their complexity limits interpretability, which were essential in clinical settings like ED. While these models excel in accuracy, their “black-box” nature can reduce clinician trust and hinder decision-making [50]. However, SVM might require more computational power and fail at the task of interpretability, that was an important factor in ED settings [51].

In such scenarios, an RF, GNB, or KNN model may be a more accessible and easily interpretable solution for clinical decision-making. For example, a GNB model, as in this study, might perform best in outputting overall prediction accuracy and falls short in terms of results when considering CTAS level precision and recall, but the reported practical feasibility and ease of use in real-world clinical practice have to be assessed as well [52].

The structure and size of our dataset al.lowed KNN and SVM to attain competitive results, despite the fact that RF and boosting algorithms frequently perform well in medical data applications. We speculate that the hyperplane optimization in SVM and the distance-based nature of KNN were especially well-suited for this classification challenge, where feature overlapped was minimal. The scaling applied during preprocessing likely benefited KNN and SVM, which are sensitive to feature scaling, while tree-based models are not. Additionally, oversampling techniques used to address class imbalance may have introduced patterns more easily captured by KNN and SVM. Simpler models like KNN and SVM may have been less prone to overfitting, given the dataset’s size and complexity. These results suggest that model selection should align with data characteristics, and future research will explore advanced feature engineering, larger datasets, and hybrid approaches to further optimize model performance. A comparative examination of these models, along with possible explanations for their surprisingly good performance, will be covered in future work [53–55].

In the current study, while the Gaussian Naive Bayes (GNB) model achieved the highest overall accuracy, its limitations are evident in misclassifications at the extreme CTAS levels. This was due to the GNB’s assumption of normally distributed features, which may not hold for skewed data or outliers, leading to misclassifications in extreme cases. Additionally, GNB’s assumption of feature independence may fail to capture important correlations between medical indicators, especially for extreme triage levels. Future research could explore feature engineering or hybrid models to address these limitations and improve performance for extreme cases [56, 57].

The interpretability of machine learning models, notably SVM and RF were a substantial barrier to clinical use. These algorithms produce accurate predictions, but lack transparency, making it difficult for doctors to grasp the reasons behind triage judgements. This lack of interpretability may undermine trust in ML-based systems and impede their inclusion into real-world emergency care processes [10]. Recent advances in Explainable Artificial Intelligence (XAI), such as LIME and SHAP, offer potential answers by providing case-specific explanations and assigning priority ratings to particular aspects. These strategies have the potential to boost physician confidence and make it easier to integrate machine learning models into ED triage systems [10]. Future research should focus on constructing hybrid models that balance predictive performance with transparency, to ensure that ML-driven triage systems fit with clinical reasoning and decision-making.

Our machine learning models outperformed prior research in CTAS triage prediction, as evidenced by their accuracy, precision, recall, and F1-score (Table 4). The results demonstrated that our models, notably GNB (98.4% accuracy) and SVM (high F1-score consistency), outperform previous research using comparable assessment measures. Compared to Hall et al. (2023) [30] and Chen et al. (2023) [32], our models had greater overall accuracy and recall, implying a better capacity to properly categorised CTAS levels. While Porto (2024) [45] demonstrated high results with XGBoost and RF, our findings indicate that Gaussian Naïve Bayes (GNB) and Support Vector Machine (SVM) have equivalent or superior prediction capabilities. This demonstrated the efficacy of our strategy in using retrospective CTAS data to enhance triage prediction.

**Table 4:** Comparing our study’s findings with previous research demonstrated the strength of our ML models in CTAS triage prediction

	Models Used	Accuracy	Precision	Recall	F1-score
Current Study (KAUH Dataset)	SVM, RF, GNB, LGBM	98.4% (GNB)	97.6%	97.2%	97.5%
Hall et al. (2023) [30]	ML Acuity Score Model	93.5%	92.8%	91.6%	92.2%
Chen et al. (2023) [32]	Deep Neural Network (DNN)	95.1%	94.3%	93.7%	94.0%
Porto (2024) [10]	XGBoost, RF	96.2%	95.7%	94.9%	95.3%

The precision of triage judgments was the critical for patient outcomes. Undertriaged can delay necessary care, increasing morbidity and mortality, while overtriaged leads to resource waste and longer wait times. In current study the model demonstrated strong performance, accurately identifying critical cases with a sensitivity of 97.69% (95% CI: 97.22–98.10%). Specificity was 96.36% [20].

The study obtained good prediction accuracy but has potential for improvement. The investigation was based on a retrospective data sample from patients’ ED visits, which may have been influenced by bias or data input problems. Future studies should take into account forthcoming data and employ bigger data sets for model building and validation. The study’s primary goal was to evaluate various machine learning methodologies, rather than to execute the model in a hospital setting. Future studies should look at obtaining massive datasets from several sources.

## Conclusion

The study found that machine learning models can enhance triage accuracy in pediatrics ED, possibly improving resource allocation and patient care. However, given the study’s retrospective nature and single-institution dataset, the findings should be regarded with caution. More multi-centered research and prospective validation are required before these models can be broadly used or utilized to drive policy choices. More research would be needed into the broader impact of machine learning on regional or national emergency care plans, including real-time model deployment, external validation, and the incorporation of explainable AI frameworks. Enhanced resource allocation using insights gained from this study can help ED administrators optimize the utilization of resources, such as medical staff, equipment, and space, thereby improving the efficiency of patient management and reducing wait times and overcrowding. This research can provide valuable evidence to inform policy and decision-making at the local, regional, or national level by utilizing predictive big data modelling techniques. The experimental results indicate that ML models can achieve high results in predicting triage based on the CTAS levels, learn the basic features and patterns of the relation between them, and successfully predict the class (CTAS), especially considering the SVM’s performance. However, testing other ML applicability and the contribution of other ML models is important in contexts where transparency and the ability to explain it’s paramount. Therefore, we recommend that this comprehensive framework be studied on larger KAUH datasets that include not only children’s data but also adult records. In addition, we examined the potential of synthetic data generation or augmentation of medical records to address dataset size issues and potential class imbalance.

### Possible applications in the future

Machine learning models for ED triage face limitations due to a single institution dataset and a retrospective training process. These limitations may limit the applicability of the models and introduce biases in real-time decision-making. Future studies should use larger, multi-centered datasets to increase model robustness and external validity. Hybrid models, combining classical machine learning with deep learning, may improve forecast accuracy and interpretability. Advanced feature engineering may improve triage variable selection. Integrating ML models into real-time ED triage procedures and testing them with prospective research is crucial for determining their therapeutic impact. Creating explainable AI frameworks for triage scenarios may increase clinician trust and accelerate model adoption in emergency care. Future research could include packaging predictive models in a KAUH system or integrating them with existing triage systems in hospitals. Deep Learning models can enhance the predictive output of triage operations, and integrating large language model capabilities can provide explainable output to staff. Integrating these models into clinical practice could include embedding them in electronic health record systems for real-time triage help. Future integration of these models into clinical practice might include embedding them in electronic health record (EHR) systems to provide real-time triage help. Furthermore, creating clinician-friendly dashboards with interpretable results, incorporating alarm systems for high-risk situations, and performing pilot studies in emergency settings may improve practical applicability. Collaboration with healthcare providers will be vital for ensuring seamless adoption and improving patient outcomes.

## Electronic supplementary material

Below is the link to the electronic supplementary material.

Supplementary Material 1

## Data Availability

The raw dataset are not publicly available to preserve individuals’ privacy in accordance with the ethical guidelines set forth by the Saudi National Committee of Bioethics (NCBE) and local data protection laws in Saudi Arabia. Any use of this data will require appropriate anonymization to ensure the privacy and confidentiality of individuals involved.
