# Supplementary material for: Predicting triage of pediatric patients in the emergency department using machine learning approach
Source: Int J Emerg Med. 2025 Mar 10;18:51. doi: 10.1186/s12245-025-00861-z (PMC11892228; doi:10.1186/s12245-025-00861-z)
Supplement: Supplementary file 1 — Supplementary Material 1 [file 12245_2025_861_MOESM1_ESM.docx]

**Supplementary Tables:**

Next, we analyzed the models’ performance on the classification of each CTAS level by examining other evaluation metrics such as Precision, Recall, and F1-score. Supplementary Tables 1-6 present the evaluation results for each model, and we notice that the best-performing model in terms of individual-level prediction is the GNB model, with a mean F-score of 97.

**STable 1: SVM performance evaluation of individual CTAS level prediction.**

| **CTAS Level** | **Precision** | **Recall** | **F1-score** |
| --- | --- | --- | --- |
| CTAS 1 | 0.96875 | 1 | 0.984127 |
| CTAS 2 | 0.970588 | 0.978814 | 0.974684 |
| CTAS 3 | 0.986869 | 0.991878 | 0.989367 |
| CTAS 4 | 0.987421 | 0.945783 | 0.966154 |
| CTAS 5 | 1 | 0.857143 | 0.923077 |

**STable 2: RF performance evaluation on individual CTAS level prediction**

| **CTAS Level** | **Precision** | **Recall** | **F1-score** |
| --- | --- | --- | --- |
| CTAS 1 | 0.775 | 1 | 0.873239 |
| CTAS 2 | 0.883268 | 0.961864 | 0.920892 |
| CTAS 3 | 1 | 0.95533 | 0.977155 |
| CTAS 4 | 0.91954 | 0.963855 | 0.941176 |
| CTAS 5 | 0.538462 | 1 | 0.7 |

**STable 3: LGBM performance evaluation on individual CTAS level prediction**

| **CTAS Level** | **Precision** | **Recall** | **F1-score** |
| --- | --- | --- | --- |
| CTAS 1 | 0.885714 | 1 | 0.939394 |
| CTAS 2 | 0.885496 | 0.983051 | 0.931727 |
| CTAS 3 | 1 | 0.967513 | 0.983488 |
| CTAS 4 | 0.988024 | 0.993976 | 0.990991 |
| CTAS 5 | 0.875 | 1 | 0.933333 |

**STable 4: KNN performance evaluation on individual CTAS level prediction**

| **CTAS Level** | **Precision** | **Recall** | **F1-score** |
| --- | --- | --- | --- |
| CTAS 1 | 0.933333 | 0.903226 | 0.918033 |
| CTAS 2 | 0.960699 | 0.932203 | 0.946237 |
| CTAS 3 | 0.97123 | 0.993909 | 0.982439 |
| CTAS 4 | 0.993421 | 0.909639 | 0.949686 |
| CTAS 5 | 1 | 0.857143 | 0.923077 |

**STable 5: GNB performance evaluation on individual CTAS level prediction**

| **CTAS Level** | **Precision** | **Recall** | **F1-score** |
| --- | --- | --- | --- |
| CTAS 1 | 0.96875 | 1 | 0.984127 |
| CTAS 2 | 0.962656 | 0.983051 | 0.972746 |
| CTAS 3 | 0.989848 | 0.989848 | 0.989848 |
| CTAS 4 | 0.993711 | 0.951807 | 0.972308 |
| CTAS 5 | 0.875 | 1 | 0.933333 |

**STable 6: DTC performance evaluation on individual CTAS level prediction**

| **CTAS Level** | **Precision** | **Recall** | **F1-score** |
| --- | --- | --- | --- |
| CTAS 1 | 0.775 | 1 | 0.873239 |
| CTAS 2 | 0.883268 | 0.961864 | 0.920892 |
| CTAS 3 | 1 | 0.95533 | 0.977155 |
| CTAS 4 | 0.91954 | 0.963855 | 0.941176 |
| CTAS 5 | 0.538462 | 1 | 0.7 |
